# Extended-spectrum β-Lactamase–producing *Enterobacteriaceae*, Central African Republic

**DOI:** 10.3201/eid1205.050951

**Published:** 2006-05

**Authors:** Thierry Frank, Guillaume Arlet, Valerie Gautier, Antoine Talarmin, Raymond Bercion

**Affiliations:** * Institut Pasteur de Bangui, Bangui, Central African Republic;; † Université Pierre et Marie Curie (Paris VI), Paris, France;; ‡ Hôpital Tenon AP-HP, Paris, France

**Keywords:** Extended-spectrum, β-lactamase, Central African Republic, letter

**To the Editor:** Since the early 1980s, extended-spectrum β-lactamases (ESBLs) have been the largest source of resistance to broad-spectrum oxyimino-cephalosporins among *Enterobacteriaceae* (*1*). Molecular analysis techniques suggest that many ESBLs are derived from mutations in TEM-1, TEM-2, and SHV-1 β-lactamases and that these ESBLs can hydrolyze the extended-spectrum cephalosporins (particularly ceftazidime) and aztreonam (*1*). Members of a new group of ESBLs have been recently identified (*1*). Among them, CTX-M–type ESBLs are rapidly expanding and are derived from chromosomal class A β-lactamases of *Kluyvera* spp (*1**,**2*). The CTX-M enzymes are not related to TEM or SHV enzymes, as they share only 40% identity with these ESBLs (*2*).These ESBLS are usually characterized by a higher level of resistance to cefotaxime than ceftazidime, except for CTX-M-19 (*2*). Most organisms that harbor ESBLs are also resistant to other classes of antimicrobial drugs, such as aminoglycosides, fluoroquinolones, chloramphenicol, and tetracyclines (*1**,**2*).

Reports concerning the existence of ESBL-producing *Enterobacteriaceae* in sub-Saharan Africa are scarce. We therefore conducted a study in the Central African Republic to determine the frequency of ESBLs in *Enterobacteriaceae* isolated at the Institut Pasteur de Bangui and to characterize their *bla*_TEM_, *bla*_SHV_, and *bla*_CTX-M_ genes.

From January 2003 to March 2005, all *Enterobacteriaceae* isolated from human specimens at the Institut Pasteur de Bangui were screened for ESBLs. Antimicrobial drug susceptibility was determined by using the disk diffusion method (Bio-Rad, Marnes la Coquette, France) on Mueller-Hinton agar (MHA) and interpreted according to the recommendations of the Comité de l'Antibiogramme de la Société Française de Microbiologie (CA-SFM) (http://www.sfm.asso.fr). ESBL-producing *Enterobacteriaceae* were selected by the following criteria: susceptibility to cefoxitin; decreased susceptibility to cefotaxime (30 μg), ceftazidime (30 μg), or cefepime (30 μg) (zone diameter <21 mm); and enhanced susceptibility in the presence of clavulanic acid by the double disk synergy test (*3*). For suspected ESBLs, the MICs of broad-spectrum cephalosporins were determined by using the agar dilution method.

We screened 450 *Enterobacteriaceae* for ESBLs during the study. We isolated and identified 17 (4%) ESBL-producing strains (Table). These strains were associated with urinary tract infection, pneumonia in an AIDS patient, wound infection, vaginal or intestinal colonization, and ear infection. We found that 11 isolates were more resistant to cefotaxime (MIC >256 μg/mL) than to ceftazidime (MIC <128 μg/mL), which suggests CTX-M–type enzymes. *Enterobacteriaceae* strains that harbor ESBLs were frequently associated with resistance to aminoglycosides and ciprofloxacin (Table).

**Table:** Characteristics of extended-spectrum β-lactamase–producing Enterobacteriaceae in Bangui, Central African Republic

Strain†	Patient hospitalized	Results of sequencing			MICs of β-lactams (μg/mL)*							
		*bla*_CTX-M_	*bla*_SHV_	*bla*_TEM_	AMC	CTX	CAZ	CRO	FEP	CPO	ATM	Resistance
*K. pneumoniae* 022	N		SHV-2a	TEM-1	16	16	16	16	8	8	2	KGT
*K. pneumoniae* 043	Y		SHV-12	TEM-1	16	16	256	32	8	8	256	KGTNC
*K. pneumoniae 106*	Y	CTX-M-15		TEM-1	8	256	128	256	64	256	64	None
*K. pneumoniae* 047	Y		SHV-2a	TEM-1	64	16	16	16	8	16	32	None
*E. coli* 272	Y	CTX-M-15		TEM-1	32	256	128	256	128	256	128	KGTNC
*E. coli* 065	Y	CTX-M-15		TEM-1	20	256	128	256	64	256	128	C
*E. coli* 047	N	CTX-M-15		TEM-1	16	256	32	256	32	128	64	KGTC
*E. coli* 010	N	CTX-M-15		TEM-1	32	256	128	256	128	256	256	KGT
*E. coli* 073	N	CTX-M-15		TEM-1	16	256	128	256	128	256	128	KGTC
*E. coli* 059	Y	CTX-M-15		TEM-1	19	256	128	256	8	256	256	C
*E. coli* 064	N	CTX-M-15		TEM-1	128	256	128	256	64	256	64	C
*E. coli* 070	N	CTX-M-15		TEM-1	128	256	128	256	64	256	128	C
*E. coli* 054	N	CTX-M-15		TEM-1	128	256	32	256	64	256	32	KGTC
*E. coli* 026	N	CTX-M-15		TEM-1	32	256	64	256	128	256	256	KGTC
*E. cloacae* 081	Y		SHV-12	TEM-1	32	16	256	16	0.125	1	256	KGTN
*E. cloacae* 106	Y		SHV-12	TEM-1	128	16	256	16	32	8	256	KGT
*E. aerogenes* 014	Y	CTX-M-3	SHV-12	TEM-1	128	256	256	256	32	256	128	KGTN

*AMC, amoxicillin + clavulanic acid (2 μg/mL); CTX, cefotaxime; CAZ, ceftazidime; CRO, ceftriaxone; FEP, cefepime; CPO, cefpirome; ATM, aztreonam; K, kanamycin; G, gentamicin; T, tobramycin; N, netilmicin; C, ciprofloxacin. †On *Klebsiella pneumoniae* strains, polymerase chain reaction and sequencing for *bla*_SHV_ genes were studied on *Escherichia coli* transconjugant or electroporant.

The conjugal transfer of the resistance determinants was carried out in trypticase soy (TS) broth with rifampin-resistant *Escherichia coli* J53-2 as the recipient. Mating broths were incubated at 37°C for 18 h. Transconjugants were selected on MHA plates containing rifampin (250 μg/mL) and cefotaxime (2.5 μg/mL). If conjugal transfer failed, plasmid DNA was extracted from donors with the Qiagen Plasmid Mini Kit (Qiagen, Courtaboeuf, France); 20 μL of *E. coli* DH10B cells were transformed with plasmid DNA by electroporation according to the manufacturer's instructions (Bio-Rad). Transformants were incubated for 1.5 h at 37°C in TS broth and then plated on MHA plates supplemented with 2.5 μg/mL cefotaxime.

Plasmid-encoded β-lactamase genes were detected on clinical isolates and their tranconjugants or transformants by polymerase chain reaction with oligonucleotide primer sets specific for the *bla*_TEM_, *bla*_SHV_, and *bla*_CTX-M_ genes (*4*). PCR assays were performed on total DNA extracted by using the commercial Qiagen DNA Mini Kit. The 3 β-lactamase genes were detected in different clinical isolates (Table). PCR results showed that the strains were harboring >2 different types of β-lactamases.

Plasmid-encoded β-lactamase genes were characterized by direct DNA sequencing with PCR primers. The nucleotide sequences were analyzed by the BLASTN (nucleotide basic local alignment search tool) program. For ESBLs, the gene types (SHV-2a, SHV-12, CTX-M-15, and CTX-M-3) were identified from different *Enterobacteriaceae* (Table). Only 1 strain (*Enterobacter aerogenes*) harbored 2 different ESBLs (CTX-M-3 and SHV-12). We identified TEM-1 and CTX-M15 enzymes, which are the most prevalent β-lactamases detected in our strains.

ESBL-producing *Enterobacteriaceae* have been previously described in South Africa (*5*), Kenya (*6*), Senegal (*7*), Cameroon (*8*), Tanzania (*9*), and Nigeria (*10*). As described in these countries, we found that CTX-M-15, SHV-2a, and SHV-12 were the most prevalent enzymes. CTX-M-15, the most recently described ESBL type, is particularly common in Bangui and seems to be closely related to *E. coli*, as was previously observed in Tanzania (*9*). This finding is also the first report of CTX-M-3 in sub-Saharan Africa.

Multidrug resistance profiles involving non–β-lactam antimicrobial drugs coselected these ESBL-producing isolates. We suggest that the misuse of antimicrobial drugs in the Central African Republic and the migratory flux of regional populations could result in emergence and selection of these ESBL phenotypes in the community. We could not establish a relationship between the different strains isolated in hospitalized and ambulatory patients. Because of the implications for treating such infections, particularly in developing countries, the spread of ESBL-producing *Enterobacteriaceae* merits close surveillance in the Central African Republic.
